# Noninferiority of selective and micropulse laser trabeculoplasties: a meta-analysis and systematic review

**DOI:** 10.1007/s10103-025-04450-7

**Published:** 2025-04-17

**Authors:** Ahmed Al-Wizni, Mohammad Saleki, Choon Yit Preston Dean Lee, Patrick Hurt, Ahmed Adan, Abdulmalik Alsaif, Ahmed Nahrawy

**Affiliations:** 1 Broomfield Hospital, Chelmsford, UK; 2 Bolton NHS Foundation Trust, Bolton, UK; 3 Queen Mary University of London, London, UK; 4 Stockport NHS Foundation Trust, Stockport, UK; 5 Kings College Hospital, London, UK; 6 King Khaled Eye Specialist Hospital, Riyadh, Saudi Arabia; 7 Western Eye Hospital, London, UK

**Keywords:** SLT, MLT, Glaucoma, Laser trabeculoplasty

## Abstract

Chronic open-angle glaucoma (COAG) affecting over 70 million people globally, is one of the leading causes of irreversible blindness. To lower intraocular pressure (IOP) in COAG, common treatments include Selective Laser Trabeculoplasty (SLT) and Micropulse Laser Trabeculoplasty (MLT). This systematic review and meta-analysis evaluates the efficacy, safety, and clinical outcomes of both treatments in managing open-angle glaucoma. A systematic review and meta-analysis following PRISMA guidelines, with searches across MEDLINE, EMBASE, EMCARE, CINAHL, and the Cochrane Central Register of Controlled Trials (CENTRAL) up to July 2024. Five studies, including 462 eyes from COAG patients, met the inclusion criteria. Primary outcomes were the success rate (defined as ≥ 20% IOP reduction) and mean IOP reduction. Secondary outcomes included adverse events, medication use, and requirement for glaucoma surgery. No significant difference was observed between both treatments in success rates (OR = 0.91, 95% CI: 0.61–1.36, *P* = 0.64). Mean IOP reduction at 3–6 months (MD = 0.46 mmHg, 95% CI: -0.43–1.36, *P* = 0.31) and 6–12 months (MD = 0.72 mmHg, 95% CI: -0.22–1.65, *P* = 0.13) showed no significant differences. Medication requirements, post-laser IOP spikes, and requirement for further surgery were similar between both treatments (OR = 2.31, 95% CI: 0.75–7.15, *P* = 0.15). SLT and MLT are both effective and safe for COAG, showing no significant differences in efficacy or safety over one year. MLT’s cooling cycles may reduce IOP spikes, but further studies are needed to confirm long-term outcomes. Clinicians can choose between treatments based on patient-specific preferences and needs.

## Introduction

Glaucoma is one of the leading causes of irreversible blindness in the world, affecting more than 70 million people globally [1]. Glaucoma is broadly classified into open-angle and angle-closure glaucoma, with open-angle accounting for 80% of cases in the United States [2]. Chronic open angle glaucoma (COAG) is a spectrum of conditions characterised by optic neuropathy and degeneration of retinal ganglion cells, cupping of the optic disc and progressive vision loss, starting from the peripheral vision to then affecting the central vision [2]. Often called the ‘thief of sight’, as up to 30–50% of ganglion cells may be lost prior to any noticeable visual field loss. This can be ascribed to the ability of the brain to adapt to losses in peripheral vision [3, 4]. Given that studies indicate more than 50% of patients are unaware of their disease, the actual number of affected individuals may be significantly higher than reported figures, as many cases remain undiagnosed until later stages [5].

One study estimated that there was an increase of 2.4 million cases of bilateral blindness caused by chronic open angle glaucoma between the years 2010 and 2020 [1]. Given this significant increase in prevalence in recent years, the availability of effective management options to halt disease progression is of paramount importance. The only modifiable risk factor for disease progression is elevated intraocular pressure (IOP), therefore treatment options typically work by reducing pressures. Management options for chronic open angle glaucoma can be divided into medical therapy, laser treatment and surgery.

First-line medical treatment for COAG involves eye drops that reduce IOP and slow disease progression. These eye drops vary in their mechanisms of action, effectiveness and side effect profile. Generally, eye drops work by either increasing uveoscleral and trabecular outflow of aqueous, or by decreasing aqueous production [6].

There are various laser treatments available for chronic open angle glaucoma. Selective laser trabeculoplasty (SLT) is a commonly used laser technique involving the use of a frequency-doubled Nd: YAG laser, delivering short pulses of energy to the trabecular meshwork to increase aqueous outflow. It is referred to as ‘selective’ as it specifically targets pigmented cells in the trabecular meshwork and does not damage adjacent structures. Micropulse laser trabeculoplasty (MLT) is the latest laser technology introduced in 2008, which also works by specifically targeting pigmented cells in the trabecular meshwork (TM) with either a 532 nm, 577–810 nm near-infrared diode laser, allowing it to target the pigmented cells of the TM in a similar way but allows for a cooling period in between a pulsed, rather than continuous laser beam [7]. This cooling cycle offers a theoretical advantage of fewer complications, given its prevention of cellular morphological changes in the TM and structural damage in surrounding tissue [8].

Despite the increasing adoption of MLT, clinical evidence directly comparing its efficacy and safety with SLT remains limited [9–13]. Thus, debate continues over whether MLT offers comparable IOP-lowering efficacy across different follow-up periods. Understanding these differences is critical for guiding clinicians toward optimal treatment choices for individual patients.

This systematic review and meta-analysis aim to fill this knowledge gap by directly comparing SLT and MLT in terms of success, IOP reduction, medication burden and adverse events. By synthesizing data from multiple studies, we hope to provide a clearer understanding of how these two laser treatments compare, helping clinicians make more informed treatment decisions based on patient needs, safety profiles, and clinical outcomes.

## Methods

### Study selection

A systematic review and meta-analysis were conducted in accordance with the Preferred Reporting Items for Systematic Reviews and Meta-Analyses (PRISMA) guidelines [14]. Titles and abstracts for all search results were reviewed independently by two authors. Full texts were obtained for all relevant studies and eligible articles were selected for our review. All discrepancies in study selection were resolved by discussion between authors.

### Eligibility criteria

All studies which compared SLT and MLT in patients with glaucoma were included. MLT was the intervention group of interest, while SLT was the comparator. Inclusion criteria were as follows: clinical studies comparing SLT and MLT, including randomised control trials, studies where full-text were available, publication in peer-reviewed journals. No age, sex/gender, or morbidity status restrictions were used; nor language restrictions were set. Exclusion criteria were: abstracts only, case reports, review studies and single-arm observational studies with no comparator publications.

### Primary outcomes

The primary outcomes were success of procedure, post-laser mean IOP and mean reduction in IOP. Success was defined most commonly as ≥/>20% reduction in IOP post-laser as this was the most commonly reported success parameter in the included studies. A further breakdown of how each study reported success is included in the results section.

### Secondary outcomes

The secondary outcomes included mean percentage decrease in IOP, mean number of antiglaucoma medications, post-laser IOP spike at 1 h and number of patients requiring glaucoma surgery despite laser trabeculoplasty. Additionally, adverse events such as inflammation, peripheral anterior synechiae (PAS) formation, long-term endothelial effects, and post-laser IOP spikes where data was available.

### Literature search strategy

Electronic databases including MEDLINE, EMBASE, EMCARE, CINAHL, and the Cochrane Central Register of Controlled Trials (CENTRAL) were searched by two authors independently. The last search was conducted on July 1st, 2024. Thesaurus headings, search operators, and limits in each of the above databases were adapted accordingly. In addition, World Health Organization International Clinical Trials Registry (http://apps.who.int/trialsearch/), ClinicalTrials.gov (http://clinical-trials.gov/), and ISRCTN Register (http://www.isrctn.com/) were searched for details of ongoing studies. The search terminologies included “Selective Laser Trabeculoplasty”, “Micropulse Laser Trabeculoplasty”, “SLT”, “MLT” and “glaucoma”. Adjuncts such as “and” as well as “or” were used to combine terms. The bibliographic lists of the relevant studies were also reviewed to extend the screening for eligible articles.

### Data extraction and management

A Microsoft Excel (Microsoft Corp.) data extraction spreadsheet was amalgamated in keeping with the Cochrane data collection form for intervention reviews. A pilot test of the spreadsheet was conducted extracting data from random articles and adapting it where necessary. Data extraction was done independently by two authors. Study characteristics were collected using a predetermined data sheet. Characteristics extracted included: study design, level of evidence, demographic characteristics and clinical outcome measures. Corresponding authors of all eligible studies were contacted via email to seek any missing data relevant to our review.

### Data synthesis

A meta-analysis was performed for outcomes reported by no fewer than 3 studies. The mean difference (MD) was used to assess continuous variables for the intervention and control groups. Data analysis was done using Review Manager 5.3 and Microsoft Excel. Fixed and random effect models were used. Reported outcomes were given in forest plots at 95% CIs. Heterogeneity was assessed via the Cochran Q test (χ^2) and quantified inconsistency by calculating I2. This value was interpreted as follows: 0–25% (low heterogeneity), 25–75% (moderate heterogeneity), and 75–100% (high heterogeneity).

### Methodological quality and risk of bias assessment

Articles matching the inclusion criteria were assessed for quality and risk of bias by two authors independently. Domains assessed included selection bias, performance bias, detection bias, attrition. RoB was assessed using the Cochrane Risk of Bias tool for the RCT study [15]. The Risk of Bias in Nonrandomized Studies of Interventions tool was used for non-randomised studies [16]. Visual presentations of risk of bias assessment were created with the Robvis tool [17].

## Results

### Literature search

**Fig. 1:**
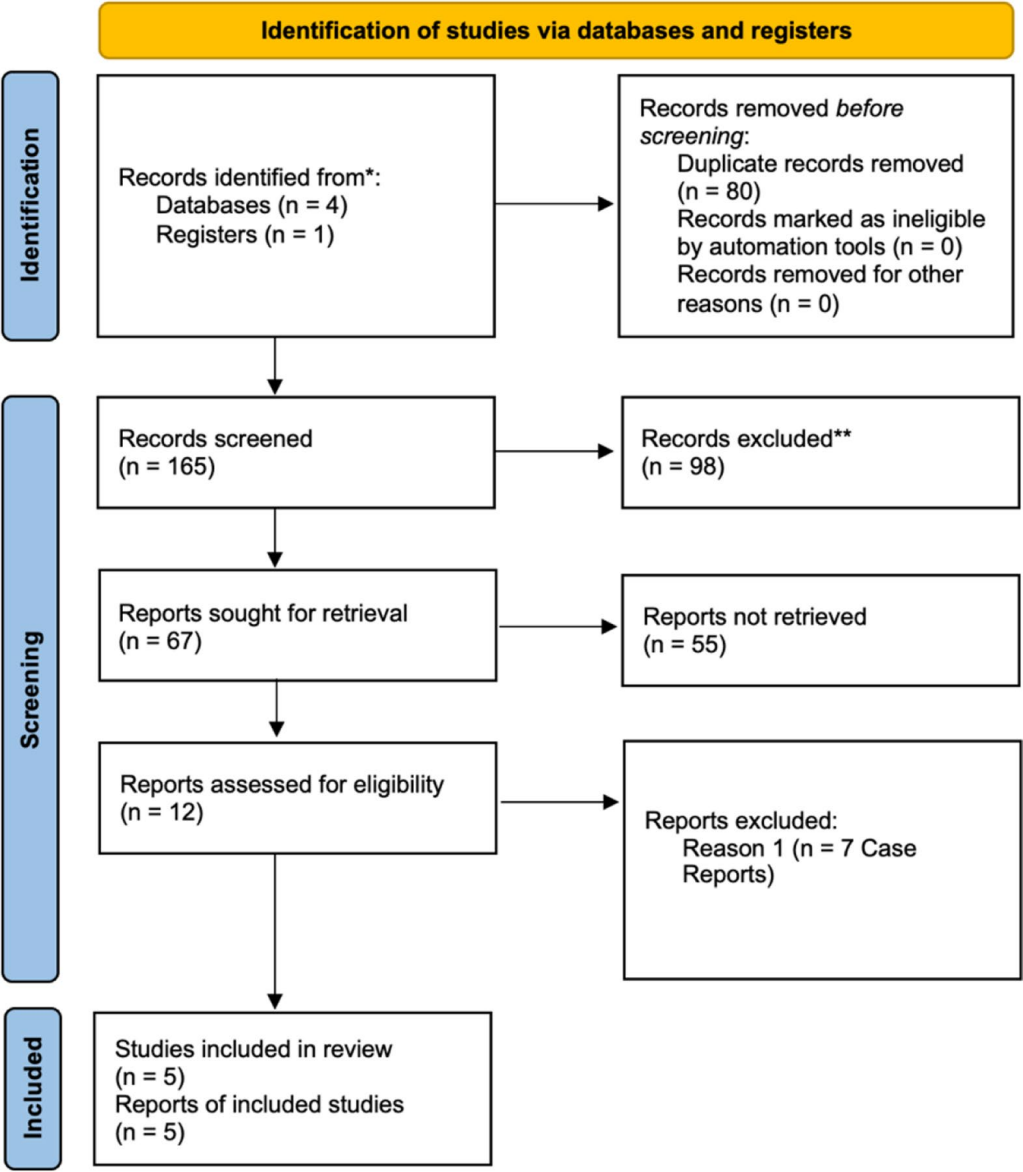
Prisma Flow Diagram for this Meta-analysis

The search strategy yielded 165 studies (Fig. 1). Following the removal of 80 duplicate records, 98 studies were excluded after the title and abstract screening. From 67 reports sought for retrieval, 55 were not retrieved due to the following reasons: full-text unavailability, inappropriate interventions. After full-text review by authors AA and MS, 12 reports were assessed for eligibility, and 7 were excluded for being case reports. Ultimately, 5 studies met the eligibility criteria for inclusion in the review: Hirabayashi et al., Sun et al., Abramowitz et al., Pimentel et al., De Leon et al.

### Description of studies

Table 1 summarises the baseline characteristics of the 5 included studies, which included 4 retrospective cohort studies and 1 prospective cohort study. Of which 4 were multicentre studies and 1 was single centre, with a total of 462 eyes from X number of patients. The studies were standardised for patient population and design, comparing SLT and MLT for patients with open angle glaucoma undergoing laser trabeculoplasty. Table 2 outlines key methodological controls across the studies, including how each addressed baseline IOP, glaucoma severity, and prior glaucoma treatments—factors that may influence treatment outcomes and heterogeneity.

Hirabayashi et al. conducted a single-centre retrospective cohort study which included 50 SLT treated eyes and 50 MLT treated eyes for open angle glaucoma between July 2017 to May 2018, to identify and compare the two treatments at 6 months follow up. IOP was measured at baseline and at 6 months. Success rates were recorded, as well as complications and IOP spike.

Sun et al. conducted a single centre retrospective cohort study comparing SLT and MLT for open angle glaucoma patients undergoing primary laser trabeculoplasty between July 2012 to October 2017. This included 85 eyes treated with SLT and 43 eyes with MLT. Patients were followed up for up to two years. Baseline IOP and mean IOP at each interval were recorded, as well as complications, medication number and success rates.

Abramowitz et al. conducted a single-centre retrospective comparative cohort study which compared the efficacy, safety and tolerability of SLT vs. MLT for treatments in open angle glaucoma patients, of which 31 eyes were treated with SLT and 38 with MLT. Patients were followed up at multiple time intervals for up to a year, where IOP reduction measurements were recorded. Success rate and complications were also recorded.

Pimentel et al. conducted a multi-centre comparative study to compare the efficacy of SLT and MLT for primary open angle glaucoma patients between March 2018 to February 2020. 52 eyes were treated with SLT and 46 with MLT and patients were followed up at multiple intervals for up to a year. Mean IOP reduction from baseline was recorded at each interval as well as success rates, complications and medication number.

De Leon et al. conducted a single centre prospective clinical trial to compare both SLT and MLT for the treatment of open angle glaucoma and ocular hypertension between March 2016 to September 2016. 38 eyes treated with SLT and 29 with MLT. Patients were followed up 1 week, 1 month then 3 months later. Mean IOP pressure was recorded at each interval, IOP reduction, medication number and success rate were also recorded.

**Table 1:** Characteristics of included studies [9–13]

Study (Year)	Journal, Country	Study Design	Dates study was done	Region of study (City, Country)	Intervention: Control (MLT :SLT) *n* (eyes)	Age	Male: Female	Ethnicity	Laser settings (SLT: MLT)	Baseline IOP (MLT vs. SLT)	Follow up	Definition of success
Hirabayashi et al. 2019	Clinical ophthalmology	**Single centre Retrospective comparative study**	**Jul 2017 - May 2018**	**Washington**,** DC [USA]**	**50:50**	**67.8 +-9.9** **(MLT)** **69.0 +-10.8** **(SLT)**	**41:59**	**41 (White)**,** 9 (Other)** **(MLT)** **47 (White)**,** 3 (Other)** **(SLT)**	**1000 mW**,** 15% duty cycle**,** 300 ms duration**,** 300 micrometre spot size** **(MLT)** **532 nm freq doubled Q-switched Nd: YAG with 3ns pulse and 400 micrometer spot size**,** variable power 0.6 and 1.4 mJ**,** titrated to visible microbubble over 360 degrees of angle** **(SLT)**	**18.3 +- 5.09** **(MLT)** **17.3 +- 4.83** **(SLT)**	**6 months**	**≥ 20% IOP reduction or ≥ 1 medication reduction without additional IOP lowering procedures**
Sun et al., 2021	Clinical ophthalmology	**Single centre Retrospective comparative cohort study**	**Jun 2012 - Oct 2017**	**San Francisco**,** [USA]**	**43:85**	**68 (63–72)** **(MLT)** **74 (71–77)** **(SLT)**	**43:48**	**15 (White)**,** 7 (Asian)**,** 4 (Hispanic)**,** 4 (Black)**,** 1 (Other)**,** 1 (Unknown)** **(MLT)** **29 (White)**,** 15 (Asian)**,** 6 (Hispanic)**,** 4 (Black)**,** 2 (Other)**,** 3 (Unknown)** **(SLT)**	**1000 mW**,** 15% duty cycle**,** 300 ms duration**,** 300 micrometre spot size** **(MLT)** **532 nm freq doubled Q-switched Nd: YAG with 3ns pulse and 400 micrometer spot size**,** variable power 0.5 and 1.6 mJ** **(SLT)**	**18.0 (16.4–19.5)** **(MLT)** **18.2 (17.2–19.3)** **(SLT)**	**1–2 years**	**≥ 20% from baseline or met prespecified target IOP with no additional glaucoma medication or subsequent glaucoma intervention**
Abramowitz et al., 2018	Clinical ophthalmology	**Single centre Prospective comparative cohort study**	**Aug 2013– Jul 2017**	**Washington**,** DC [USA]**	**38:31**	**66.1** **(MLT)** **67.6** **(SLT)**	**28:41**	**N.A.**	**1000 mW**,** 15% duty cycle**,** 300 ms duration**,** 300 micrometre spot size** **(MLT)** **532 nm freq doubled Q-switched Nd: YAG with 3ns pulse and 400 micrometer spot size**,** variable power 0.6 and 2.0 mJ** **(SLT)**	**18.26** **(MLT)** **16.85** **(SLT)**	**1 year**	**≥ 20.0% IOP decrease**
Pimentel et al., 2023	Lasers in medical science	**Multi centre Retrospective comparative study**	**Mar 2018 - Feb 2020**	**Salvador**,** Bahia**,** [Brazil** **Lauro de Freitas**,** [Brazil]** **Salvador**,** [Brazil]** **Campinas**,** [Brazil]**	**46:52**	**62.7 +-4.1** **(MLT)** **61.4 +-3.2** **(SLT)**	**42:56**	**N.A.**	**1000 mW**,** 15% duty cycle**,** 300 ms duration**,** 300 micrometre spot size** **(MLT)** **532 nm freq doubled Q-switched Nd: YAG with 3ns pulse and 400 micrometer spot size**,** variable power 0.6 and 1.4 mJ**,** titrated to visible microbubble over 360 degrees of angle** **(SLT)**	**23.8 +- 1.5** **(MLT)** **23.4 +- 2.4** **(SLT)**	**1 year**	**IOP ≤ 21 mmHg and ≥ 20% reduction from baseline IOP without additional medications**,** new laser session**,** or glaucoma surgery**
De Leon et al., 2017	International journal of medical research & health sciences	**Single centre Prospective clinical trial**	**Mar 2018 - Sep 2016**	**Mexico City**,** [Mexico]**	**29:38**	**71.84 +-10.16** **(MLT)** **70.08 +-7.79** **(SLT)**	**39:38**	**N.A.**	**1000 mW**,** 15% duty cycle**,** 300 ms duration**,** 300 micrometre spot size** **(MLT)** **180° of the TM (superior or inferior)**,** exposure time prefixed 3n/s**,** spot size 400 μm**,** power beginning with 0**,**6 mJ/pulse**,** and increasing it 0**,**1 mJ/pulse** **(SLT)**	**19.76 ± 4.23** **(MLT)** **19.53 ± 4.03** **(SLT)**	**3 months**	**> 20% in IOP**

**Table 2:** Study controls for Glaucoma severity, previous Glaucoma treatments, and baseline IOP [9–13]

Study (Year)	Control for Baseline IOP	Control for Glaucoma Severity	Control for Previous Glaucoma Treatment
Hirabayashi et al. 2019	Yes (Stratified success rates based on baseline IOP ≤ 18 mmHg vs. >18 mmHg)	Yes (Mild, Moderate, Severe)	No
Sun et al., 2021	Yes (Sensitivity analysis comparing baseline IOP from two prior visits, one prior visit, or the laser day visit)	No	Yes (Excluded prior laser trabeculoplasty, laser cyclophotocoagulation and glaucoma surgery)
Abramowitz et al., 2018	Yes (Pretreatment IOP calculated as the average of up to three preceding visits)	No	Partially (included patients on maximally tolerated medical therapy but did not specify prior treatments)
Pimentel et al., 2023	Yes (Reported mean baseline IOP for SLT vs. MLT groups)	No	Yes (Excluded patients with prior laser, filtering, or recent ocular surgery (within 6 months), ocular trauma, or steroid use)
De Leon et al., 2017	Yes (Collected baseline IOP data before laser treatment)	No	Partially (Some patients may have been on topical hypotensive treatments)

### Quality assessment

**Fig. 2:**
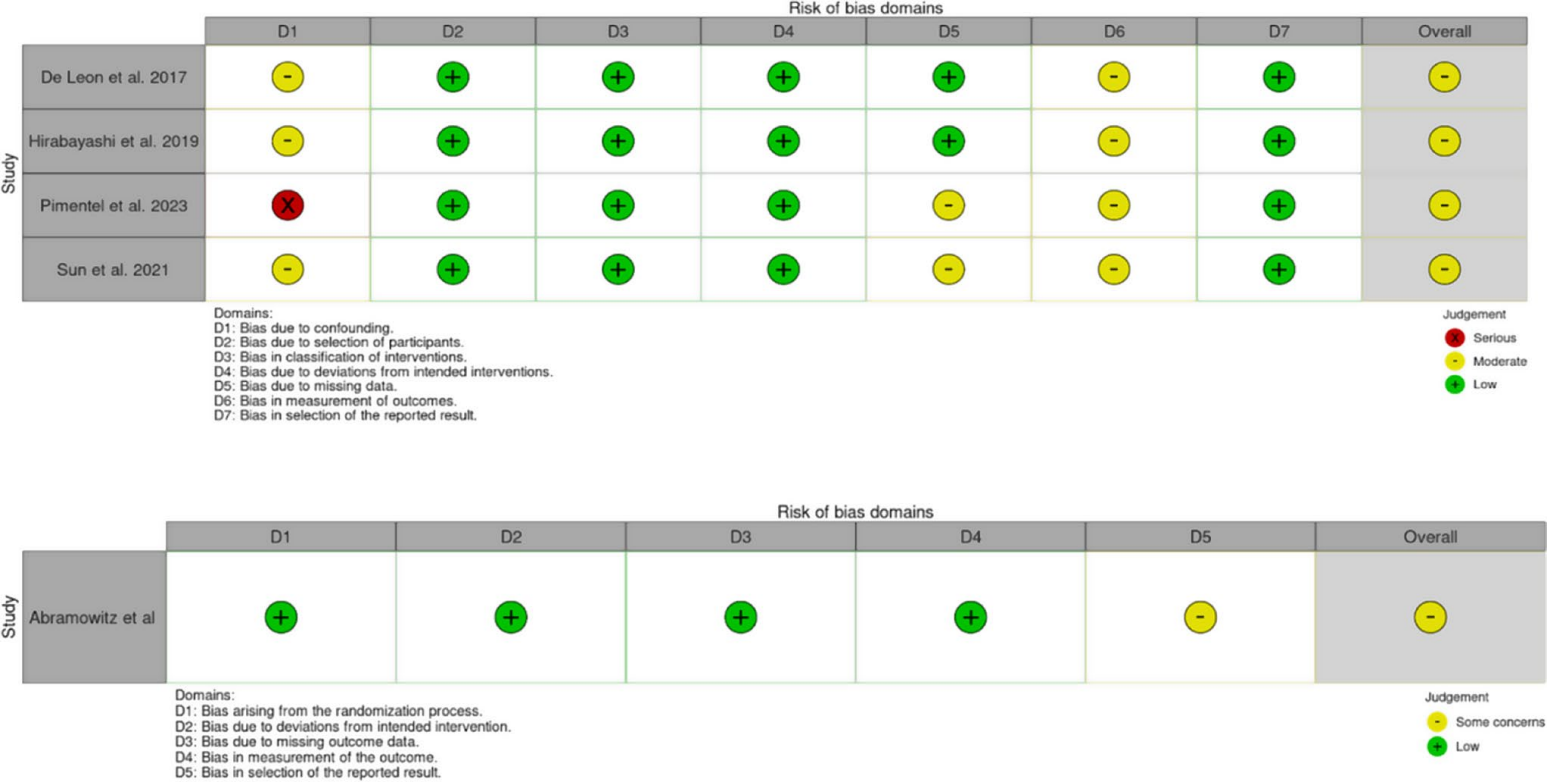
Risk of bias assessment; **A**) Robvis traffic light plot for ROBIN-I risks of bias analysis of non-randomised trials **B**) Robvis traffic light plot for ROB-2 risks of bias analysis of the randomised trial

Overall, all studies included were shown to be of good quality with acceptable risk of bias, as assessed by the ROBIN-I and ROB-2 classification systems. Two separate systems were used according to their randomisation as per Cochrane guidelines. The results of these analysis are shown in Figs. 2.

### Primary outcomes

#### Success

**Fig. 3:**
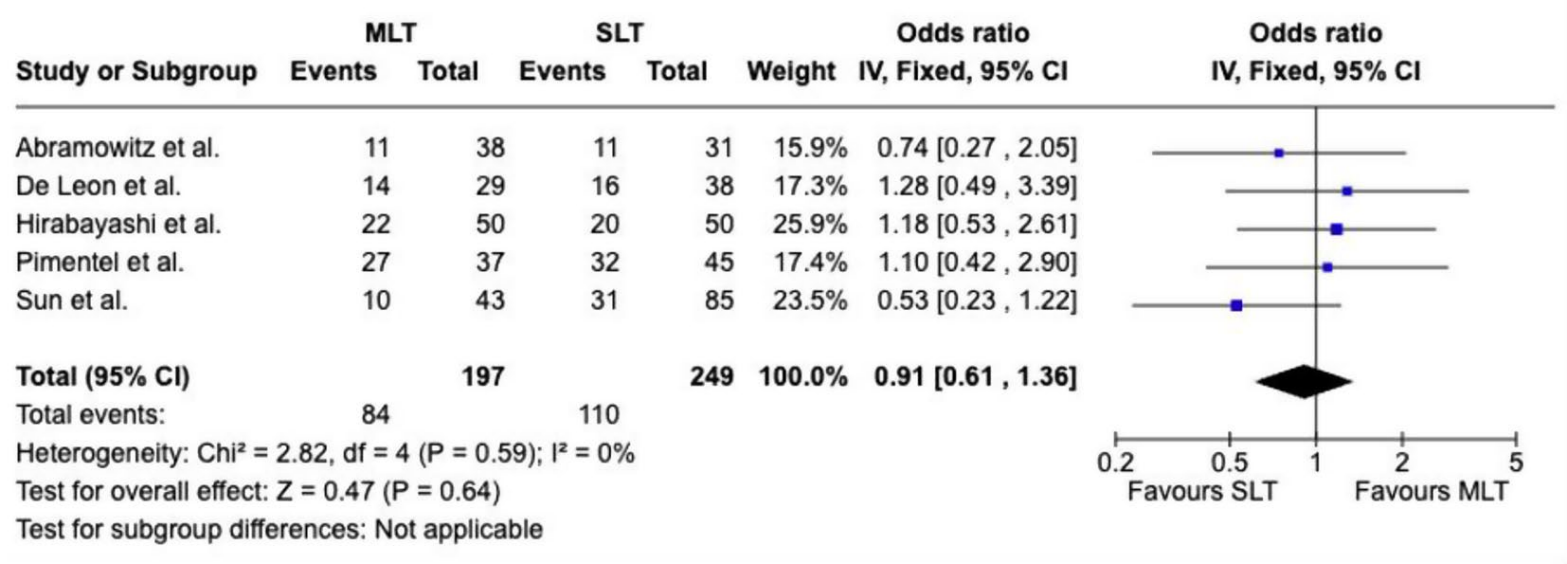
Forest plot of success rates of SLT vs. MLT [9–13]

Success was reported in five studies with a combined total of 446 eyes. No statistically significant difference was found between the MLT and SLT (Odds ratio (OR) = 0.91, 95% confidence interval (CI) 0.61 to 1.36, *P* = 0.64). A low level of heterogeneity among the studies was noted (I² = 0%, *P* = 0.59); Fig. 3.

#### Intraocular pressure (IOP)

Mean IOP was compared between SLT and MLT groups at different time intervals.

**Fig. 4:**
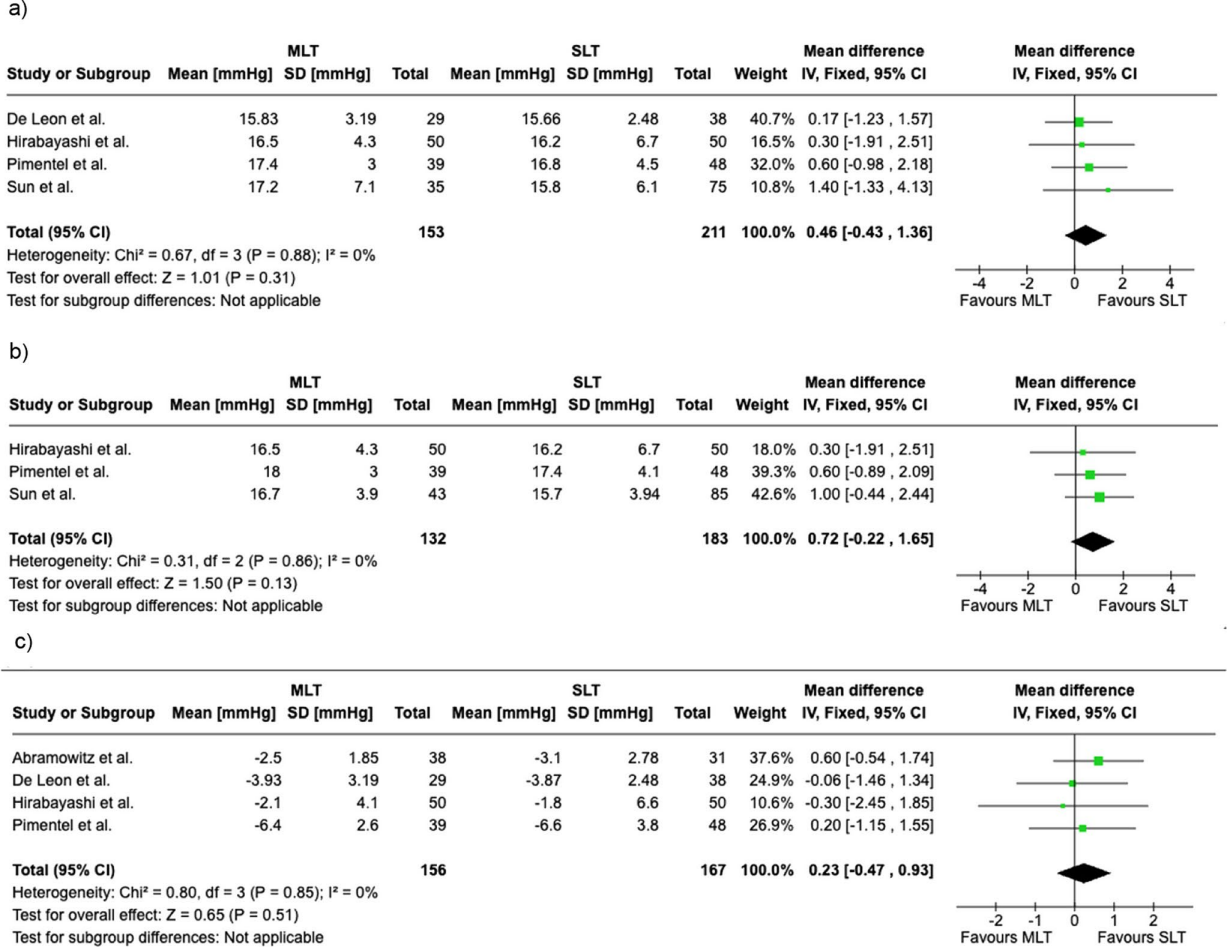
Forest plot relating to intraocular pressure outcomes comparing SLT vs. MLT; **a**) Mean IOP at 3-6 months post SLT & MLT, **b**) Mean IOP at 6-12 months post SLT & MLT, **c**) mean IOP reduction at 3-6 months post SLT & MLT [9–13]

Mean IOP at 3–6 months was reported in four studies with a total of 364 eyes. No statistically significant difference was found between SLT and MLT during this time interval (mean difference (MD) of 0.46, 95% CI: -0.43 to 1.36, *P* = 0.31. The heterogeneity among the studies was low (*P* = 0.88, I² = 0%); Fig. 4A.

Mean IOP at 6–12 months was reported in three studies and included a total of 315 eyes. The mean difference was found to be 0.72 mmHg (95% CI: -0.22 to 1.65), showing no significant difference (*p* = 0.13). The heterogeneity among these studies was also low (*P* = 0.86; I² = 0%); Fig. 4B.

The study by Abramowitz et al. was excluded from the pooled mean IOP analyses due to missing standard deviation values, which precluded its inclusion in the meta-analysis. However, its findings were reviewed and discussed qualitatively. De Leon et al. only reported data up to 3 months follow up and hence was not included in the 6–12 months analysis. Mean IOP reduction at 3–6 months was reported by four studies with a total of 323 eyes. No statistically significant difference was found between SLT and MLT (MD = 0.23, 95% CI: -0.47 to 0.93,*P* = 0.51). The heterogeneity among the studies was also low (*P* = 0.85, I2 = 0%). Findings are shown in Fig. 4C.

### Secondary outcomes

#### Number of IOP Lowering medications

The number of medications required was reported in four studies with a total of 364 eyes. No statistically significant difference was found between the two interventions (MD = 0.25, 95% CI: 0.00 to 0.51, *P* = 0.05). A low level of heterogeneity was noted (I²= 0%, *P* = 0.98); Fig. 5A.

#### IOP Spike at 1 h post laser

IOP spike 1-hour post-treatment was reported in four studies following SLT and MLT with a total of 379 eyes. No statistically significant difference was noted between the two groups (OR = 0.42, 95% CI: 0.16 to 1.05, *P* = 0.06). A moderate level of heterogeneity was noted (I² = 29%, *P* = 0.24); Fig. 5B.

#### Glaucoma surgery

**Fig. 5:**
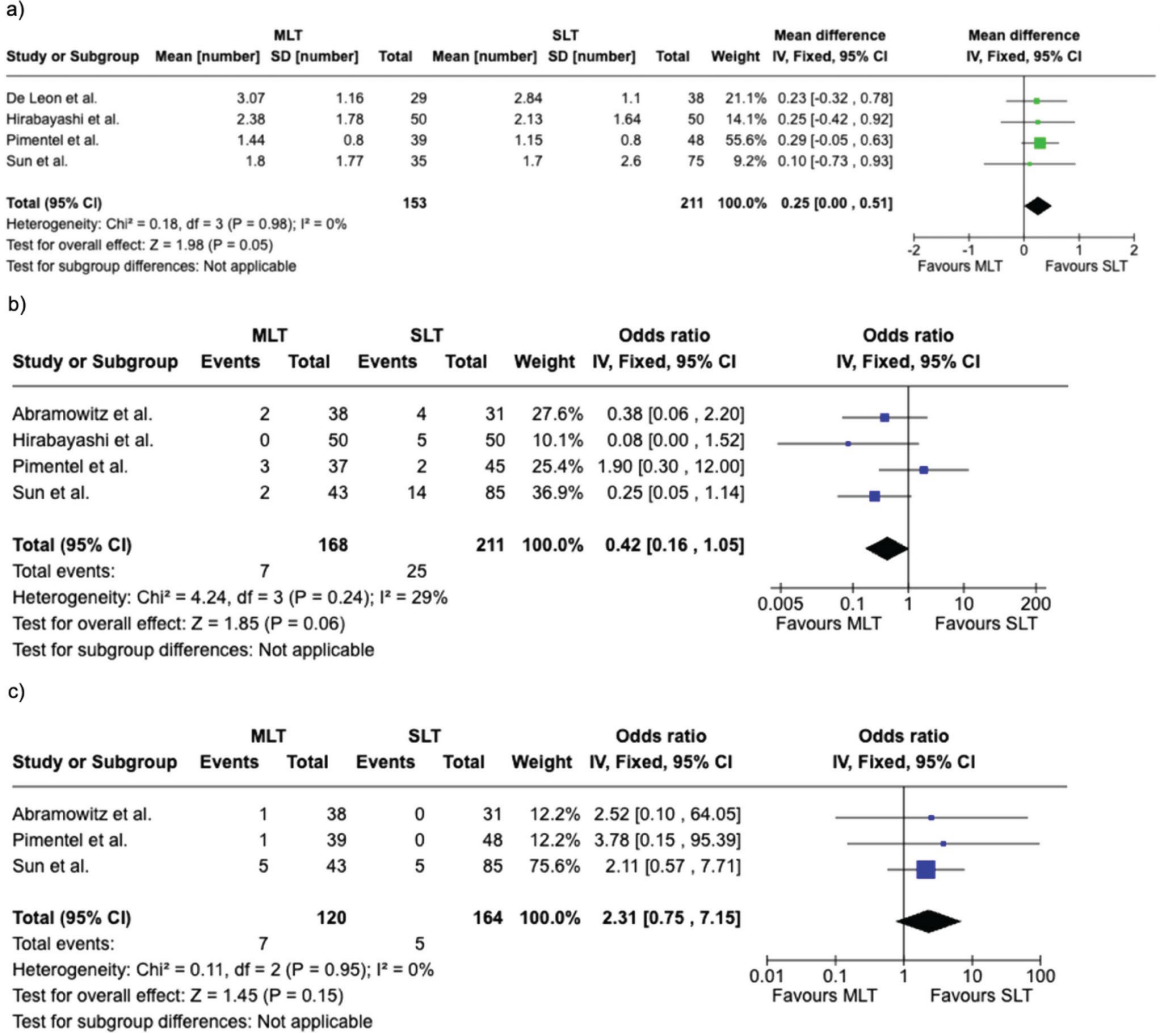
Forest plot comparing secondary outcome measures. **a**) Number of IOP lowering medications for SLT and MLT, **b**) 1-hour IOP spike following SLT and MLT, **c**) need for glaucoma surgery post-laser [9–13]

### Adverse events

Among the five studies included, only Abramowitz et al. reported that no group experienced anterior chamber inflammation upon examination at the 1-week follow-up. Additionally, the MLT group reported less pain both during and after the procedure (*P* = 0.005), suggesting reduced post-laser inflammation compared to SLT. The remaining studies did not assess or report complications such as PAS formation, long-term endothelial effects, or persistent inflammation following treatment.

## Discussion

This meta-analysis evaluated the success rates and efficacy of MLT and SLT in reducing IOP for patients with glaucoma. The findings indicated that there is no statistically significant difference between MLT and SLT in achieving a > 20% reduction in IOP, with both treatments demonstrating comparable success rates. The mean IOP reduction at both short-term (3–6 months) and long-term (6–12 months) follow-up periods showed no substantial difference between the two treatments, suggesting that MLT and SLT are comparable to each other in lowering IOP at the 12-month mark. Additionally, the number of glaucoma medications required post-treatment did not differ significantly between the two groups, indicating similar effectiveness in reducing dependency on medications.

The incidence of adverse events, such as IOP spikes post-treatment, was also comparable between MLT and SLT, further supporting the similarity in safety profiles of both laser treatments. While our meta-analysis suggests a slight favouring of SLT in terms of patient outcomes, this difference was not statistically significant. Moreover, the long-term efficacy of SLT compared to MLT in repeated laser trabeculoplasty treatments remains ambiguous, as none of the five studies extended beyond a 12-month period. Despite this, the low heterogeneity among the studies still strengthens the reliability of our findings.

Most studies comparing SLT and MLT are similar in their complication rates, and capabilities in their IOP reduction potential among the investigated patient groups [18–22]. The only exception was that, in this predominantly African American and Hispanic population, MLT demonstrated a greater decrease in mean IOP percentage from baseline, although the sample size was very small, which is a limitation [23]. Moreover, regarding complications, a study also displayed almost no IOP spikes for Primary Open Angle Glaucoma (POAG) patients undergoing MLT, which was the most prevalent complication following SLT, while having a similar 1-year post procedure failure rate [21, 24, 25]. Despite the merits of MLT, there has been a retrospective study suggesting that MLT’s success rate might be problematic, with few patients achieving significant IOP reductions, although variability in glaucoma severity and different surgeons performing the procedure could have influenced the outcomes [26].

Considering the slight difference in IOP reducing capabilities between the two treatment methods, it may thus be helpful to look towards the pathway for which various countries are undertaking. Whilst glaucoma eye drops remain the mainstay for first-line treatment in many countries, multiple studies, including the Laser in Glaucoma and Ocular Hypertension (LiGHT) trial, have shown SLT’s effectiveness in controlling IOP and its favourable safety profile as a primary treatment [27–29]. The strength of these studies has led the UK’s National Institute for Health and Care Excellence (NICE) to designate SLT as the preferred first-line treatment over medication. A 2024 report by the American Academy of Ophthalmology’s Ophthalmic Technology Assessment Committee has also found level 1 evidence supporting SLT as an appropriate primary intervention strategy [30–32].

Since the margin of difference between IOP-reducing capabilities are similar between both techniques, the investigation of cost would likely be a crucial factor in determining the long-term applications of each laser. As the Current Procedural Code (CPT) for both SLT and MLT is the same, the savings passed on to the patients are thus similar as well. However, from the cost perspective of a physician, the utility of the IQ 532 nm™ laser (Iridex Corporation, Mountain View, CA, USA) extends beyond MLT and can be used for stimulating the retinal pigment epithelium, retinal photocoagulation, laser peripheral iridoplasty, and laser suturolysis [8, 11]. These supplementary utilities could subsequently boost the cost effectiveness of a practise, potentially passing the savings onto patients. Another incentive for making laser therapy a first-line treatment is also the fact that SLT is more cost-effective than most brand-name glaucoma drops within one year of the procedure and is presumably cheaper than most generic medications within 13–40 months [10].

Ultimately, this meta-analysis found no significant difference in efficacy in lowering IOP, indicating that MLT can be considered a reliable treatment comparable to SLT. However, our study revealed intriguing insights that could guide future research in this area. Abramowitz et al. highlighted that secondary glaucoma showed higher success rates with laser trabeculoplasty compared to primary open-angle glaucoma [9]. This disparity in success rates based on glaucoma subtype underscores the importance of future research focusing on tailoring treatment strategies based on glaucoma subtype which could enhance the overall effectiveness of laser trabeculoplasty in managing open-angle glaucoma. Sun et al. also noted that MLT was associated with less pain and inflammation compared to SLT suggesting that beyond IOP reduction, factors such as patient comfort and postoperative inflammation may vary between MLT and SLT, warranting further investigation into the underlying mechanisms [10].

This meta-analysis has several limitations that should be acknowledged when interpreting the results. One notable limitation is the slightly different measures of success used across the included studies, with some defining success as ≥ 20% IOP reduction, while others included medication reduction or target IOP achievement. However, despite these slight variations, the success criteria remain largely standardized at a ≥ 20% IOP reduction, making comparisons between studies still valid. Additionally, variations in follow-up durations could have introduced bias and affected the comparability of outcomes. The relatively small sample size—encompassing only 462 eyes across five studies—may limit the robustness and generalisability of our findings. While these were the only studies meeting our predefined inclusion criteria, this reinforces the need for larger, high-quality studies in this area. Differences in baseline characteristics, such as pretreatment IOP levels, may have also influenced the overall results. Another key limitation is the relatively short follow-up periods in most studies, with the majority assessing outcomes only up to 12 months. Consequently, the long-term durability of treatment effects remains uncertain. Future randomized controlled trials (RCTs) with extended follow-up periods are needed to evaluate sustained efficacy. Furthermore, most studies focused solely on IOP reduction and medication burden, without assessing functional outcomes such as visual field progression or quality of life and while statistical heterogeneity was low, the lack of subgroup or sensitivity analyses in most studies limits confidence in this finding and underscores the need for larger, more robust trials. Incorporating these parameters in future RCTs would provide a more comprehensive understanding of the real-world clinical impact of MLT and SLT. Lastly, we also acknowledge the wide confidence intervals observed (e.g., OR = 0.91, 95% CI: 0.61–1.36), which reflect the modest sample size and contribute to statistical uncertainty, though this is mitigated by consistent findings across timepoints and low heterogeneity.

Despite these limitations, this meta-analysis provides valuable insights into the efficacy and safety of MLT and SLT in managing open-angle glaucoma. The findings suggest that both laser trabeculoplasty techniques offer comparable IOP-lowering effects and safety profiles, making them viable options for treatment. Although MLT has not been around for very long, making long-term follow-up data limited, the available evidence suggests it is still a useful measure of success. The need for standardized protocols and outcome measures remains critical to ensure more robust and comparable results. Future research should also focus on addressing these limitations by conducting longer follow-up studies to assess the sustained efficacy and impact of these treatments over multiple years. Addressing these limitations in future research will be essential for refining treatment guidelines and optimizing patient care.

Data Sharing Statement:

All data needed to evaluate the conclusions in the paper are present in the paper.

## Data Availability

All the data from the five studies can be found in the following links https://pmc.ncbi.nlm.nih.gov/articles/PMC6585400/https://pmc.ncbi.nlm.nih.gov/articles/PMC6124459/https://link.springer.com/article/10.1007/s10103-023-03771-9https://www.researchgate.net/publication/380459060_SELECTIVE_LASER_TRABECULOPLASTY_VS_MICROPULSE_LASER_TRABECULOPLASTY_FOR_THE_TREATMENT_OF_OPEN_ANGLE_GLAUCOMA_AND_OCULAR_HYPERTENSION
